# Child-to-Parent Violence Among Adolescents: A Preliminary Analysis of Its Association with Sociodemographic Variables, Dating Violence, and Antisocial Traits

**DOI:** 10.3390/children12020243

**Published:** 2025-02-18

**Authors:** Alba Espuig, Laura Lacomba-Trejo, Francisco González-Sala

**Affiliations:** 1 Facultat de Psicologia i Logopèdia, Universitat de València, 46010 València, Spain; alesa3@alumni.uv.es; 2 Department of Developmental and Educational Psychology, Facultat de Psicologia i Logopèdia, Universitat de València, 46010 València, Spain; francisco.gonzalez-sala@uv.es

**Keywords:** adolescents, dating violence, psychopathy, antisocial and law-violating behaviors, child-to-parent violence

## Abstract

Introduction: Child-to-parent violence (CPV) is influenced by factors such as sex, age, dating violence, psychopathy traits, and antisocial and law-violating behaviors. This study explores how these variables relate to aggression towards parents, identifying key explanatory factors. Methods: This research engaged 136 Spanish adolescents aged 15–18 (mean age = 16.47; 51% female). Assessments included the Conflict in Adolescent Dating Relationships Inventory (CADRI) for dating violence, the Psychopathy Content Scale (P-16) for psychopathy, the Antisocial and Criminal Behavior Scale in Adolescents (ECADA) for antisocial and law-violating behaviors, and the Conflict Tactics Scales (CTS2) for CPV. Analyses of associations included linear regression and qualitative comparative analysis (QCA). Results: Females exhibited higher levels of verbal violence, less delinquency, and more frequent CPV towards mothers. Psychopathy, antisocial and law-violating behaviors, and exposure to verbal violence were correlated with filial aggression. Violence towards mothers was associated with older age, female sex, verbal violence exposure, and psychopathy (47% variance explained), while violence towards fathers was linked to younger age and psychopathy (28% variance explained). QCA results indicated that specific combinations of having experienced violence and psychopathic traits contribute to CPV towards both parents. Conclusions: These findings highlight the importance of addressing psychological and sociodemographic risk factors for CPV. Prevention efforts should focus on reducing exposure to violence, identifying at-risk adolescents, and implementing targeted interventions to promote family well-being.

## 1. Introduction

According to the World Health Organization [1], adolescence, spanning the ages 10 to 19, marks the transition from childhood to adulthood. This is a critical period for emotional and physical development, making early coping strategies and affective relationships essential for healthy adaptation to changes, optimal development, and the challenges characteristic of this stage. The potential for optimal development during adolescence is significantly influenced by experiences in earlier life stages [2]. However, adolescent development is not only shaped by past experiences, but also by the broader socio-cultural context in which an adolescent is currently embedded [3,4]. Proximal factors, such as family dynamics, peer relationships, and school environment, play a crucial role in shaping emotional, cognitive, and behavioral development [5,6,7,8]. Additionally, distal influences, including cultural norms, socioeconomic conditions, and broader societal structures, contribute to opportunities and constraints that impact adolescent well-being and adjustment [9,10]. These interacting influences underscore the importance of considering both past experiences and present environmental conditions when examining adolescent developmental trajectories.

Considering the various criminological theories on the progression of delinquent behavior during adolescence, which suggest an increase starting around age 14, peaking at ages 16–17, and subsequently declining in incidence, adolescence emerges as a critical period for identifying variables relevant to predicting violence [11,12,13]. In this sense, antisocial behavior is defined by actions that go against the integrity of others, violate legal or social norms, and are frequent, intense, or severe [14]. These behaviors include aggression, breaking social or legal norms, drug abuse, and pre-antisocial and law-violating behaviors, understood as actions outside societal norms [15].

Therefore, psychopathy is understood as a syndrome characterized by antisocial, impulsive, manipulative, and callous behavior [16], along with a lack of empathy and grandiosity. Despite its similarities to antisocial personality disorder, psychopathy includes narcissistic, borderline, and histrionic personality traits, and is not considered a mental health disorder [17]. The expression of psychopathic traits differs between men and women [18], which may underlie the disparities in the perpetration of various types of offenses [19]. Women tend to exhibit fewer physically violent behaviors, instead demonstrating a higher prevalence of verbal aggression, whereas men are more frequently associated with acts of physical violence [20,21,22]. This gender-related distinction is partially linked to a higher incidence of psychological aggression by women towards their mothers, whereas males exhibit a greater frequency of physical aggression towards both parents [23]. The relationship between psychopathy and antisocial behaviors, including criminal acts and parental violence, is well established across the life cycle, particularly in adolescents [24], with psychopathy serving as a significant predictor of violent behaviors [25]. This relationship has also been observed by Shaffer et al. [26] in the context of dating violence. As with other forms of violent behavior, gender differences are evident in the typology of these actions. Specifically, within adolescent romantic relationships, women are more likely to engage in verbal aggression, whereas men are more frequently involved in severe physical aggression [27].

In this sense, child-to-parent violence (CPV) encompasses physical, psychological, or economic abuse by children or adolescents towards their parents, aiming to gain control or power over them, and can be referred also as filial or son violence [28]. Studies consistently show that exposure to family violence is a key risk factor for the development of CPV [29] as well as delinquent and pre-antisocial and law-violating behaviors [30] and dating violence [31,32,33]. Nevertheless, sociodemographic variables such as gender and age can also influence CPV. Regarding gender, a higher prevalence of females involved in filial violence among adolescents has been reported [34]. Girls tend to exhibit more verbal or psychological violence, especially towards their mothers, who are generally the primary recipients of such violence [29]. However, being male is considered a risk factor for the perpetuation of delinquent behaviors [35,36]. And it has been observed that within the context of adolescents in the judicial system, the prevalence of CPV is higher among males, whereas in community settings, gender differences are less pronounced [37]. Regarding age, a longitudinal study exhibits a general decline in CPV as adolescents grow older, with aggression peaking between the ages of 13 and 15 before decreasing during late adolescence [30]. This trajectory highlights the influence of age-related developmental dynamics on the frequency of CPV.

To the best of our knowledge, although the existing literature on the relationship between CPV, dating violence, psychopathy, and antisocial and law-violating behaviors is extensive, there are few studies that address these issues jointly. Furthermore, most of these studies predominantly employ linear prediction methodologies. Therefore, this study aims to combine the use of linear and non-linear methodologies, such as qualitative comparative analysis (QCA), to enhance the understanding of these phenomena. Given the profound impact that multiple exposures to violence—whether as a victim or a perpetrator—can exert on development during the critical period of adolescence, it is of particular interest to examine the interrelations among different forms of violence and their associations with other violent behaviors, considering both sociodemographic and psychopathological factors.

Thus, the present study aims to examine CPV towards both fathers and mothers separately, analyzing its associations with age, gender, received physical and verbal–emotional violence (dating violence), psychopathy, and antisocial and law-violating behaviors in Spanish adolescents. This objective will be addressed using two statistical methodologies: linear regressions and QCA models. Our first hypothesis (H1) posits that there will be a positive association between CPV, psychopathy, antisocial and law-violating behaviors, and dating violence. Hypothesis 2 (H2) suggests that younger age and female gender will be associated with these variables. Therefore, Hypothesis 3 (H3) posits that CPV will be linked to younger age, being female, high levels of psychopathy, high levels of antisocial and law-violating behaviors, and having experienced physical and verbal–emotional violence from a partner (dating violence).

## 2. Materials and Methods

### 2.1. Participants

This research involved 136 Spanish adolescents aged 15–18 (mean age = 16.47; 51% female). The sample was drawn from public educational institutions (57.50%) and semi-private or private institutions (42.50%), and included students in the 3rd (48.90%) and 4th (40.30%) years of compulsory secondary education and in the Baccalaureate program (10.90%) in the Valencian Community, Spain. Informed consent was obtained from both the participants and their parents.

### 2.2. Variables and Instruments

Sociodemographic variables (gender and age) were assessed using a custom scale. In addition, the following variables were assessed with questionnaires that had been previously validated in similar populations, and had demonstrated good psychometric properties in prior studies. The instruments were carefully chosen to ensure robust assessment of the key variables:

- Psychopathy: evaluated using the Psychopathy Content Scale (P-16) developed by Salekin et al. [38]. This instrument was created based on the Millon Adolescent Clinical Inventory (MACI), a 160-item self-report measure of personality and psychopathology in adolescents. In developing the P-16, Salekin et al. [38] identified the MACI items that theoretically aligned with the Revised Hare Psychopathy Checklist (PCL-R), and that also fit into Cooke and Michie’s [38] and Frick et al.’s [39] models for psychopathy. The resulting scale comprises 16 dichotomous items (true/false), that are grouped in three different subscales: callousness, egocentricity, and antisociality. The sum of the subscales can be used to obtain the total score, which is the one used in this study. In the scale construction study, the observed internal consistency was α = 0.86. And the corresponding alphas for the subscales of callousness, egocentrism, and antisociality were 0.62, 0.61, and 0.56, respectively [38]. In this study, Cronbach’s alpha was α = 0.64.
- Antisocial and law-violating behaviors: Measured using the Antisocial and Criminal Behavior Scale in Adolescents (ECADA) [15]. The scale comprises 25 dichotomous items (True/False) that evaluate the presence of antisocial and law-violating behaviors. The items are grouped into the following five dimensions:
  - Pre-antisocial and law-violating behaviors: behaviors not expressly criminal, although deviating from social norms and rules (e.g., missing school, running away from home, driving vehicles without permission or authorization, etc.).
  - Vandalistic behaviors: criminal behavior carried out on objects or property (e.g., damage to bus stops, street furniture, etc.).
  - Property offenses: criminal conduct such as robberies and robberies in different contexts and places (e.g., entrance permit in a house, building or private property) is evaluated.
  - Violent behavior: criminal conduct involving participation in assaults against persons and possession/use of weapons (e.g., carrying a weapon such as a razor).
  - Alcohol and drug use (e.g., cannabis, cocaine, or amphetamines).

The total score used in this study is obtained by the sum of the subscales. Higher scores indicate a greater presence of antisocial and law-violating behaviors. The ECADA scale has shown adequate psychometric properties, with internal consistency indices ranging from α = 0.82 to 0.86 [15,40,41]. In this study, Cronbach’s alpha was α = 0.79.

- CPV: Assessed using the Conflict Tactics Scales (CTS2)—children to parents version [42,43]. In this study, the adaptation by the Lisis Group [44] was used to assess filial violence towards parents. The scale consists of 10 items that are answered separately for the mother and the father. Responses are recorded on a five-point scale, ranging from 0 (Never) to 4 (Many times). The scale provides an overall index of child-to-parent violence, as well as scores for three specific factors: verbal violence, physical violence, and economic violence. In this study, the total score is derived by adding together the subscale scores. This version has shown adequate psychometric properties, with an internal consistency index ranging from α = 0.66 to 0.85 across subscales [44]. In this study, Cronbach’s alpha was 0.67 for the total score of violence towards the mother and 0.69 for the scale of violence towards the father.
- Dating violence: Evaluated using a brief version of the Conflict in Adolescent Dating Relationships Inventory (CADRI) [45,46]. The scale adaptation by the Lisis Group [47] was used, comprising 34 items, with 17 items pertaining to violence perpetrated and the remaining 17 items addressing violence received. The items are grouped into the three factors: relational violence, verbal–emotional violence and physical violence. Responses are recorded on a 4-point scale, ranging from 0 (never) to 3 (frequently, on six or more occasions.). In this study, to assess dating violence, only received physical violence and received verbal–emotional violence were evaluated. In the original scale, the internal consistency was α = 0.83 [45], and in the Spanish adaptation, it was α = 0.86 [46]. In this study, Cronbach’s alpha was α = 0.93.

### 2.3. Procedure

A convenience sampling approach was used, targeting adolescents from various educational centers in the Valencian Community. The selection was based on accessibility and willingness to participate, ensuring a diverse, yet manageable, sample of adolescents aged 15–18 years. After selecting assessment tools, a letter was sent to the chosen educational centers outlining the research project. The directors were then contacted by phone or email. Interviews were arranged with those who agreed to participate, where the project was detailed further, and informed consents from the regional Ministry of Education were provided, along with parental permissions for adolescents to fill out various surveys. After administering the surveys in a session lasting about an hour, responses were entered into a database for statistical analysis. Results were then extracted for interpretation and conclusion formulation. The study adhered to the ethical standards required in human research, respecting principles laid out in the updated Helsinki Declaration, including informed consent and information rights, data protection and confidentiality, non-discrimination, no charge, and the option to withdraw at any stage. Additionally, a pilot group of university students evaluated the surveys for difficulty and time required, helping to identify issues and refine the final questionnaire. This research was approved by the ethics committee of the Universitat de València (REF 2024-PSILOG-3592610).

### 2.4. Design

The study employed a non-experimental, cross-sectional approach, gathering data at one point in time to explore and describe the phenomena.

### 2.5. Analysis

Data analysis was conducted using SPSS 28.0. Descriptive statistics characterized the sample based on sociodemographic and clinical variables. Pearson correlations were used to assess relationships between dating violence (experienced physical and verbal violence), psychopathy, criminal behavior, age, and CPV towards both the mother and the father. Additionally, linear regressions and QCA models were implemented to examine parental violence towards the father and the mother, separately, considering factors such as age, gender, experienced physical violence, verbal violence, criminal behavior, and psychopathy. To complement traditional statistical analyses, fuzzy set qualitative comparative analysis (fsQCA) was employed, a method particularly suited to identifying complex interactions between factors that contribute to an outcome. This approach differs from conventional regression techniques by focusing on combinations of conditions that can lead to similar outcomes, rather than isolating the independent effect of each variable. This method requires recalibrating all variables on a scale from 0 to 1 to reflect their degree of membership in each set. Binary variables were coded as 0 (absence) and 1 (presence). Continuous variables were recalibrated automatically by the fsQCA software (4.1), setting three thresholds: 0% (low level/exclusion), 50% (intermediate level/partial membership), and 90% (high level/full inclusion) [48]. To compute total scores, scales were recalibrated and items were multiplied to derive the total score [49]. Descriptive statistics for these recalibrated conditions were generated using SPSS v28.

The fsQCA approach defines necessary conditions, which are always required for an outcome to occur, and sufficient conditions, which can produce an outcome but are not always required. QCA models quantify the explained variance and assess the proportion of cases where the model fits, termed coverage, along with indicators of model accuracy, termed consistency [49,50]. A condition is deemed necessary if its consistency is ≥0.90, and a model is considered sufficiently informative if its consistency is around or exceeds 0.74 [49,50].

Our study employed QCA to complement traditional analytical strategies as it captures complex interactions between variables, and identifies multiple configurations leading to similar outcomes. This approach enhances practical applicability by providing nuanced insights into the most relevant factor combinations, offering valuable guidance for targeted interventions. By combining QCA with linear regression, the study provides a more comprehensive understanding of the associative dynamics of filial violence.

## 3. Results

### 3.1. Descriptive Analysis and Mean Differences

Descriptive analyses showed low levels of physical (M = 0.29; SD = 1) and verbal–emotional (M = 3.85; SD = 5.51) violence received, and medium–low values of psychopathy (M = 4.02; SD = 2.55), antisocial and law-violating behaviors (M = 6.54; SD = 3.70), and CPV towards the father (M = 3.06; SD = 3.39) and the mother (M = 3.69; SD = 3.39) (Table 1).

It was observed that females experienced more verbal–emotional violence, engaged in fewer antisocial and law-violating behaviors compared to males, and exhibited more CPV towards the mother. The effect sizes were substantial. All other comparisons were not significant, and their effect sizes were small. Further details can be found in Table 1.

### 3.2. Correlational Analysis

Age was positively associated with more verbal–emotional violence received, higher levels of psychopathy, and more antisocial and law-violating behaviors. Psychopathy was positively associated with more verbal–emotional violence received and increased CPV towards both the mother and the father. CPV towards the mother was associated with more verbal–emotional violence received, higher levels of psychopathy, more antisocial and law-violating behaviors, and increased parental violence towards the father. In contrast, CPV towards the father was associated with higher levels of psychopathy and more antisocial and law-violating behaviors. More information can be found in Table 2.

### 3.3. Predictive Analysis

#### 3.3.1. Hierarchical Regression Models

The explanatory power of the variables was analyzed through a hierarchical regression model. The criterion variables were CPV against the mother and CPV against the father. The explanatory dimensions included age, gender, physical violence received, verbal–emotional violence received, psychopathy, and antisocial and law-violating behaviors. The model was structured in two steps: the first step included age, gender, physical violence received, and verbal–emotional violence received; the second step added psychopathy and antisocial and law-violating behaviors.

In the first step, the explanatory variables significantly increased the variance of CPV against the mother (ΔR^2^ = 0.18, *p* ≤ 0.001), but not against the father (ΔR^2^ = 0.05, *p* > 0.05). Age (*β* = −0.21, *t* = 3.2, *p* ≤ 0.01) and verbal–emotional violence received (*β* = 0.22, *t* = 2.92, *p* ≤ 0.01) were significantly associated with violence against the mother. Age (*β* = −0.24, *t* = −3.10, *p* ≤ 0.01) was the only significant variable associated with violence against the father in this step.

In the second step, where psychopathy and antisocial and law-violating behaviors were incorporated, the variance of CPV against the mother increased significantly (ΔR^2^ = 0.28, *p* ≤ 0.001), as did the variance of CPV against the father (ΔR^2^ = 0.23, *p* ≤ 0.001). Psychopathy was significantly associated with violence against both the mother (*β* = 0.48, *t* = 6.33, *p* ≤ 0.001) and the father (*β* = 0.52, *t* = 5.93, *p* ≤ 0.001). Antisocial and law-violating behaviors were also significantly associated with violence against the mother (*β* = 0.18, *t* = 2.30, *p* ≤ 0.05), but not against the father (*β* = −0.00, *t* = −0.01, *p* > 0.05).

Overall, the final model explained 44% of the variance in CPV against the mother (R^2^ adj = 0.44, *p* ≤ 0.001) and 25% of the variance in CPV against the father (R^2^ adj = 0.25, *p* ≤ 0.001). The Durbin–Watson statistic was 1.73 for the model examining violence against the mother and 2.06 for the model examining violence against the father, indicating no issues with autocorrelation. The variance inflation factor (VIF) values ranged between 1.09 and 1.50, indicating no collinearity issues in the regression model. Table 3 presents the detailed results of the hierarchical regression model.

#### 3.3.2. Fuzzy Set Qualitative Comparative Analysis (fsQCA)

##### Analysis of Necessary Conditions

First, the main descriptors and calibration values for the study variables are presented (Table 4). Based on the results obtained, there were no necessary conditions for high and low levels of CPV against the mother and father, as the consistency was lower than 0.90 in all cases (Table 5) [49].

##### Analysis of Sufficiency Conditions

In the sufficiency analysis, the combination of conditions that led to high and low levels of CPV against the mother and father were calculated (Table 6). According to the premise that in fsQCA, a model is informative when the consistency is around or above 0.74 [50], all models obtained were consistent.

In the explanation of high levels of CPV towards the mother, three pathways were identified, accounting for 63% of the variance (overall consistency = 0.79; overall coverage = 0.63). The first pathway accounted for 46% of the cases through the combination of being female, having experienced verbal–emotional violence from a partner, and exhibiting antisocial and law-violating behaviors. The second pathway accounted for 41% of the cases through the combination of being female, having experienced verbal–emotional violence from a partner, and having high levels of psychopathy. The third pathway accounted for 41% of the variance through the combination of being female, having high levels of psychopathy, and exhibiting high levels of antisocial and law-violating behaviors, accompanied by low levels of physical violence from a partner.

With reference to low levels of CPV towards the mother, one pathway was identified, explaining 55% of the cases (overall consistency = 0.84; overall coverage = 0.55). This pathway resulted from the combination of being older, being female, and presenting low levels of antisocial and law-violating behaviors, having experienced verbal–emotional violence, and psychopathy.

In explaining high levels of CPV towards the father, three pathways were identified, collectively accounting for 72% of the cases (overall consistency = 0.75; overall coverage = 0.72). The first pathway accounted for 55% of the variance through the combination of being female and exhibiting antisocial and law-violating behaviors and psychopathy. The second pathway also accounted for 55% of the variance through the combination of being female, exhibiting psychopathy, and having experienced physical violence from a dating partner. The third pathway, accounting for 40% of the cases, resulted from the combination of being female and having experienced verbal–emotional, but not physical, violence from a partner.

Regarding the explanation of low levels of CPV towards the father, two models were identified, collectively accounting for 61% of the cases (overall consistency = 0.79; overall coverage = 0.61). The model that explained the most (50% of the variance) did so through the combination of being older, being female, and not exhibiting antisocial and law-violating behaviors psychopathy or having experienced verbal–emotional violence from a partner. The second model accounted for 30% of the variance through the combination of being older, being female, having experienced verbal–emotional, but not physical, violence, and not exhibiting psychopathic traits.

## 4. Discussion

The present study provides a comprehensive analysis of the factors associated with CPV, emphasizing the roles of sociodemographic variables, dating violence, psychopathy, and antisocial and law-violating behaviors among Spanish adolescents. The findings offer significant insights into the complex interplay of these variables, with nuanced differences based on gender and the parental target (mother or father). The results largely align with our hypothesis, introducing novel implications for both theoretical frameworks and practical interventions.

In the first instance, Hypothesis 1 posited that a positive relationship would be found between dating violence, psychopathy, antisocial and law-violating behaviors, and CPV. The obtained outcomes substantially support this hypothesis, highlighting the associations between these variables in relation to aggressive behavior among adolescents.

Psychopathy exhibited strong correlations with CPV towards both parents. This finding is consistent with prior research suggesting that psychopathy is often linked to a wide spectrum of violent and aggressive behaviors [25]. The personality construct of psychopathy, characterized by traits such as callousness, impulsivity, and a lack of empathy, may be associated with a higher likelihood of engaging in violent behaviors, not only in peer or romantic relationships, but also within the family unit [16]. This could indicate that adolescents with elevated psychopathy scores may experience difficulties in emotional regulation and exhibit reduced empathy, which in turn might increase the probability of using violence against their parents.

Similarly, antisocial and law-violating behaviors were positively correlated with CPV, particularly towards mothers. This relationship supports the idea that antisocial tendencies—such as property offenses, substance use, and acts of vandalism—may extend beyond peer or community settings to include familial interactions [30]. Adolescents who frequently engage in such behaviors might be more likely to adopt aggression as a strategy for exerting control or managing conflicts, potentially increasing the probability of violence directed at their parents. Furthermore, based on gender socialization theory [51] this association, particularly with violence toward mothers, could be influenced by gender dynamics. Societal norms and traditional gender roles often position mothers as primary caregivers, making them more frequent targets of adolescent aggression within the household. Additionally, adolescents—especially males—may be more likely to internalize dominant or aggressive behaviors as a means of asserting control, which could be reinforced by exposure to models of hegemonic masculinity that normalize violence as a conflict resolution strategy. These dynamics highlight the need for interventions that address not only antisocial behavior, but also the underlying gendered expectations that may contribute to the disproportionate victimization of mothers in cases of CPV.

The study also found significant correlations between exposure to verbal–emotional violence in dating relationships and CPV. This finding is particularly relevant as it suggests that victimization in one relational domain may be associated with aggression in another. Adolescents exposed to verbal–emotional violence in their romantic relationships could be more likely to develop aggressive communication styles or maladaptive interaction patterns, such as hostility or coercion, which may then manifest in interactions with their parents [31]. This reinforces the notion that experiences in romantic relationships during adolescence might be interconnected with behaviors in the family domain. Interestingly, while verbal–emotional violence showed robust correlations with CPV, physical violence in dating relationships was less strongly associated. This difference may reflect variations in the psychological impact of these forms of violence. Verbal–emotional violence, which often involves sustained patterns of manipulation, humiliation, or control, could have a more enduring impact on adolescents’ behavioral and emotional development compared to isolated incidents of physical aggression.

Hypothesis 2 posited that being younger and female will be associated with these variables. Regarding age, the findings suggest a possible negative relationship, as older adolescents exhibit lower levels of FV towards both mothers and fathers. These findings align with previous research that highlights adolescence as a critical period for CPV, with younger individuals being more likely to exhibit aggressive behaviors towards their parents. Younger adolescents may find it more challenging to manage conflicts with authority figures like parents, particularly fathers, who are often perceived as enforcers of rules and boundaries. Calvete et al. [30] found that CPV tends to decrease as adolescents mature, possibly due to developmental improvements in emotional regulation and conflict resolution skills. This trajectory may reflect the transition from dependency and emotional volatility in early adolescence to greater autonomy and emotional stability in later stages.

Concerning gender, it was found that being female was associated with higher levels of received verbal–emotional violence and a greater propensity for CPV towards the mother. This aligns with the existing literature, which suggests that girls may be more likely to engage in verbal–emotional violence (in contrast to boys, who are more prone to physical violence) and to direct such aggression more frequently towards the maternal figure rather than the father [29,52,53]. These findings suggest that CPV patterns may be influenced not only by developmental trajectories but also by gendered socialization processes. Research has shown that girls are often socialized to prioritize emotional expressiveness and relational conflict [51], which could contribute to a higher prevalence of verbal aggression in contrast to physical violence, which is more common among boys. Moreover, the tendency for girls to direct aggression toward their mothers may be influenced by the traditionally stronger emotional bonds and relational interdependence between mothers and daughters, potentially leading to more intense but also more conflictive interactions [54].

Lastly, Hypothesis 3 posited that CPV would be associated with by younger age, female gender, experiences of physical and verbal–emotional violence within dating relationships, elevated levels of psychopathy, and increased antisocial and law-violating behaviors. The findings largely support this hypothesis, as they align with the relationships between variables observed in Hypotheses 1 and 2, while also revealing variations in predictors depending on the parental target (mother or father).

In the regression models, the variance explained was notably higher for CPV towards mothers (44%) compared to fathers (25%), which may indicate that these predictors have a stronger association with violence directed at mothers. Specifically, the predictors for CPV towards mothers included older age, being female, verbal–emotional violence, psychopathy, and antisocial behaviors, whereas physical violence was not a significant factor. In contrast, CPV towards fathers was primarily associated with younger age and psychopathy. The QCA offered additional insights by identifying pathways involving combinations of predictors. For CPV towards mothers, key pathways included being female, high psychopathy, and verbal–emotional violence exposure in dating relationships. Meanwhile, CPV towards fathers was associated with combinations involving psychopathy, antisocial and law-violating behaviors, exposure to dating violence (in this case including physical violence), and being female and younger. In conclusion, the results highlight the relevance of dating violence, psychopathy, and antisocial and law-violating behaviors, as they may be related to difficulties in emotional regulation and the development of maladaptive and aggressive strategies in interactions with others [16,25,26,31], as well as being younger and female, reflecting the developmental stage, maturation, and family interactions, as previously explained [29,30,53].

The results suggest that CPV cannot be analyzed without considering the gender dimension, as significant differences emerge depending on the targeted parent and the adolescent’s profile. The higher variance explained in violence towards mothers could be attributed to their role as the primary caregiver and emotional regulator, making them more frequently exposed to household conflicts [55]. Additionally, the association between being female and engaging in verbal–emotional violence towards the mother suggests the influence of gender norms that shape the expression of aggression, with girls favoring relational strategies and boys displaying more physical behaviors [51]. The relationship between psychopathy and CPV in both sexes reinforces the need for interventions focused on emotional regulation and empathy, but also on deconstructing gender roles that perpetuate the use of violence as an interaction strategy.

### 4.1. Theoretical and Practical Implications

These findings have important theoretical and practical implications. On a practical level, the results highlight the need for early interventions that focus on improving emotional regulation, empathy, and conflict resolution in adolescents, particularly those with elevated scores on psychopathy-related traits. Targeting these difficulties may help reduce CPV within families and beyond, reflecting the theoretical importance of incorporating personality traits into models of CPV.

Equally critical is the need to address verbal–emotional victimization in dating relationships, which may contribute to maladaptive communication patterns that extend into family dynamics, particularly in interactions with mothers. Prevention programs teaching healthy communication in romantic contexts could help mitigate these patterns, aligning with the theoretical understanding of how external relational stressors shape aggression within the family.

Tailored interventions based on age and gender are also essential. Younger adolescents could benefit from strategies to manage authority-related tensions with fathers, while mothers, who face higher levels of verbal–emotional aggression, require support in reducing relational strain. These measures align with the theoretical nuances of CPV, which vary depending on parental roles and dynamics.

Upon reviewing the existing literature on this subject, various systematic reviews and studies of intervention and prevention programs targeting CPV in children and adolescents highlight the limited evidence regarding the effectiveness of such programs [56,57,58]. Among the few initiatives that have demonstrated promising outcomes in reducing CPV [59,60,61], we found the Step-Up program [62], which integrates restorative practices, emotion regulation, cognitive–behavioral techniques, and skills-based training, including fostering empathy, accountability, self-soothing, respect, and effective communication. Similarly, the Early Intervention Program for CPV Situations [58] adopts a cognitive–behavioral approach incorporating systemic family therapy to promote respectful and prosocial behaviors while enhancing parent–child relationships. Additionally, the Intervention Program for Families and Minors with Abusive Behaviors [63] employs strategies such as problem-solving, self-regulation, accountability, cognitive restructuring, and communication skills training. Based on these contributions and our findings, we propose implementing interventions that address factors associated with violent behavior and psychopathy, such as fostering empathy and enhancing emotional regulation. Given the gendered nature of CPV—particularly its prevalence towards mothers—it is also essential to incorporate a gender-sensitive approach. This includes challenging traditional masculinity norms that may normalize aggression, promoting alternative conflict resolution strategies, and fostering respectful and egalitarian family dynamics. By integrating these approaches, interventions can effectively address the inter-connected factors driving CPV, thereby mitigating its prevalence, a concern further underscored by the high incidence of psychological (79.5–92%) and verbal (7.2–19.10%) violence among Spanish adolescents [29,64,65].

### 4.2. Limitations and Future Research

The present study, while offering valuable insights, is not devoid of limitations, which should be considered when interpreting the findings and provide a solid foundation for future research directions. Firstly, the sample was selected by convenience, evaluating adolescents from schools in the Valencian Community who agreed to take part in the study. In addition, the sample was drawn exclusively from educational institutions, where the prevalence of both dating violence and CPV was moderate to low. Additionally, the sample size, although adequate for preliminary observations, could benefit from expansion. While this setting allowed for the investigation of these phenomena within a general adolescent population, it may have excluded adolescents experiencing more severe or high-risk behaviors. Future research should aim to include larger and more diverse samples, incorporating participants from varied contexts such as juvenile detention centers or community-based organizations, where the incidence and dynamics of these behaviors may differ significantly. Similarly, studies could be carried out with cross-cultural samples to see if the same results are obtained and are generalizable. Expanding the sample in this way would improve the generalizability of the findings and potentially uncover additional risk factors or protective factors. Furthermore, selecting the sample randomly could improve the generalizability results.

Secondly, the cross-sectional design of this study limits the ability to establish causal relationships between the variables investigated. Although the findings support associative relationships, longitudinal studies are needed to confirm these associations and explore the temporal development of CPV. For example, longitudinal research could help identify how psychopathy traits, antisocial behaviors, or experiences of dating violence evolve over time and interact to influence the onset or escalation of CPV. For example, longitudinal studies could analyze the trajectories of different types of violence over time. This would allow for a better understanding of the variables under study, and for the design of interventions that are more tailored to the specific time in the life cycle. Such studies could also explore potential mediators and moderators, such as adverse childhood experiences, family dynamics, or peer influences, that shape these trajectories.

Another limitation lies in the reliance on self-reported data. While self-reports are instrumental in capturing adolescents’ subjective experiences, they are inherently vulnerable to biases, including social desirability and recall inaccuracies. To address this, future studies should incorporate multi-informant approaches, gathering data from parents, teachers, or other caregivers to validate and complement adolescents’ self-reports. This would not only enhance the reliability of the data, but also provide a more comprehensive perspective on the behaviors and experiences of the adolescents involved. Furthermore, Cronbach’s alpha coefficients for scales measuring key constructs, such as filial violence and psychopathy, were below the recommended threshold of ≥0.70. This may indicate some degree of measurement error or limited internal consistency within the study sample. While these scales have been widely used in prior research, caution is warranted when interpreting findings related to these constructs. Future studies should explore alternative measurement approaches, such as item response theory or confirmatory factor analysis, to further assess and refine the reliability of these scales in adolescent populations.

Finally, this study primarily focused on individual-level variables, such as psychopathy and antisocial behaviors, which, while essential, do not fully capture the broader ecological context of CPV. Future research should consider familial and community-level factors to provide a more nuanced understanding of the phenomenon. For instance, examining the role of family dynamics, including interparental violence, attachment styles, or parenting practices, could yield valuable insights. Similarly, exploring the influence of community-level factors, such as neighborhood violence, socio-economic conditions, or access to support systems, would further contextualize the findings and highlight the systemic influences on CPV. Moreover, assessments should include not only self-reports, but also hetero-informant evaluations. This approach could help mitigate certain response biases while enhancing the applicability and clarity of the results.

In conclusion, while this study makes a significant contribution to understanding the predictors of CPV, addressing these limitations in future research will advance the field further. Expanding the diversity of samples, adopting longitudinal designs, incorporating multi-informant data, and examining ecological and developmental contexts will provide deeper insights into the mechanisms driving CPV. Such advancements are essential for designing more effective interventions and prevention strategies, ultimately reducing the prevalence of CPV and fostering healthier family relationships.

## 5. Conclusions

In conclusion, this study provides valuable insights into the predictors of CPV among adolescents, emphasizing the roles of psychopathy traits, antisocial and law-violating behaviors, and experiences of verbal–emotional dating violence, as well as the influence of age and gender. The findings reveal that these factors not only exhibit significant relationships with CPV, but also serve as reliable predictors, with notable variations depending on the parental target.

These results highlight the need for practical interventions that are both multi-faceted and targeted. Programs focused on emotional regulation, conflict resolution, and healthy communication could mitigate the impact of relational victimization, while gender-sensitive and age-specific strategies would address the distinct challenges posed by developmental and relational contexts. By integrating individual, relational, and contextual factors into both theoretical models and practical strategies, this study offers a comprehensive framework for understanding and mitigating CPV. These efforts are crucial not only for reducing adolescent aggression, but also for fostering healthier family relationships and promoting the well-being of all family members.

## Figures and Tables

**Table 1:** Comparison of means between female and male adolescents in physical violence received, verbal–emotional violence received, psychopathy, antisocial and law-violating behaviors, and CPV.

		Women	Man	*t*	*p*	*d*
	M (SD)	M(SD)	M(SD)			
Physical violence received	0.29 (1)	0.31 (0.96)	0.26 (1.06)	−0.33	0.74	0.06
Verbal–emotional violence received	3.85 (5.51)	5.49 (6.56)	2.12 (3.40)	−3.79	<0.001 ***	0.64
Psychopathy	4.02 (2.55)	3.87 (2.63)	4.18 (2.47)	0.71	0.48	0.12
Antisocial and law-violating behaviors	6.54 (3.70)	5.77 (3.44)	7.36 (3.81)	2.56	0.01 **	0.44
CPV father	3.06 (3.39)	3.21 (3.23)	2.39 (3.57)	−0.55	−0.58	0.09
CPV mother	3.69 (3.39)	4.26 (3.69)	3.09 (2.95)	−2.03	0.04 *	0.35

Note: *t* = *t* value; *p* = *p* value; *d* = Cohen’s *d*; * *p* ≤ 0.05; ** *p* ≤ 0.01; *** *p* ≤ 0.001.

**Table 2:** Correlations between age, physical violence received, verbal violence received, psychopathy, antisocial and law-violating behaviors, and CPV towards both parents.

	Age	Physical Violence Received	Verbal Violence Received	Psychopathy	Antisocial and Law-Violating Behaviors	CPV Father	CPV Mother
Age	1						
Physical violence received	−0.04	1					
Verbal–emotional violence received	0.21 *	0.23 **	1				
Psychopathy	0.21 *	−0.07	0.27 **	1			
Antisocial and law-violating behaviors	0.25 **	0.06	0.28 **	0.48 **	1		
CPV father	−0.13	0.02	0.15	0.47 **	0.19 *	1	
CPV mother	−0.03	0.04	0.41 **	0.57 **	0.38 **	0.66 **	1

Note: * *p* ≤ 0.05; ** *p* ≤ 0.01.

**Table 3:** Hierarchical regression model.

Explanatory Factors	CPV Against the Mother				VIF	CPV Against the Father				VIF
	∆R^2^	∆F	*β*	*t*		∆R^2^	∆F	*β*	*t*	
Step 1	0.18 ***	7.40 ***				0.05	1.74			
Older (age)			−0.21	0.32 **	1.11			−0.24	−3.10 **	110
Women			0.17	2.31 *	1.24			−0.06	0.73	124
Physical violence received			−0.01	−0.141	1.10			0.04	0.53	1.09
Verbal–emotional violence received			0.22	2.92 **	1.43			0.03	0.35	1.43
Step 2	0.28 ***	34.20 ***				0.23 ***	20.97 ***			
Psychopathy			0.48	6.33 ***	1.38			0.52	5.93 ***	1.38
Antisocial and law-violating behaviors			0.18	2.30 *	1.50			−0.00	−0.01	1.50
Durbin–Watson	1.73					2.06				
R^2^_ajd_	0.44 ***					0.25 ***				

Note: ∆R^2^ = change on R^2^; R^2^_adj_ = R^2^_adjusted_; *β* = regression coefficient; *t* = *t* value; VIF = variance inflation factor; * *p* < 0.05; ** *p* ≤ 0.01; *** *p* ≤ 0.01.

**Table 4:** Descriptive analysis and calibration values of the fsQCA.

	Age	Physical Violence Received	Verbal–Emotional Violence Received	Antisocial and Law-Violating Behaviors	Psychopathy	CPV Against the Mother	CPV Against the Father
M	16.47	0.29	3.85	6.54	4.02	3.69	3.06
SD	0.90	1.00	5.51	3.70	2.55	3.39	3.39
Min.	15.00	0.00	0.00	0.00	0.00	0.00	0.00
Max.	18.00	7.00	30	17.00	12.00	19.00	22.00
P10	15.00	0.00	0.00	1.70	1.00	0.00	0.00
P50	16.00	0.00	2.00	6.50	4.00	3.00	2.00
P90	18.00	1.00	13.00	12.00	8.00	7.00	7.00

**Table 5:** Need analysis of CPV against the mother and the father.

	High Levels of CPV Against the Mother		Low Levels of CPV Against the Mother		High Levels of CPV Against the Father		Low Levels of CPV Against the Father	
	Cons.	Cov.	Cons.	Cov.	Cons.	Cov.	Cons.	Cov.
Older	1.00	0.03	1.00	0.03	1.00	0.03	−30.82	1.00
Younger	−30.59	1.00	−31.30	1.00	−31.06	1.00	1.00	0.03
Women	1.00	0.33	1.00	0.51	1.00	0.33	1.00	0.33
Men	−1.02	1.00	−1.04	0.68	−1.03	1.00	−1.03	1.00
High physical violence received	0.74	0.68	0.73	0.33	0.75	0.68	0.75	0.69
Low physical violence received	0.64	0.71	0.65	1.00	0.66	0.73	0.65	0.72
High verbal–emotional violence received	0.59	0.73	0.43	0.52	0.56	0.68	0.49	0.60
Low verbal–emotional violence received	0.61	0.52	0.78	0.65	0.67	0.57	0.74	0.63
High antisocial and law-violating behaviors	0.68	0.71	0.50	0.66	0.67	0.69	0.53	0.55
Low antisocial and law-violating behaviors	0.53	0.52	0.71	0.71	0.56	0.54	0.70	0.68
High psychopathy	0.67	0.77	0.48	0.53	0.67	0.75	0.50	0.56
Low psychopathy	0.59	0.54	0.79	0.70	0.61	0.55	0.78	0.70

**Table 6:** Summary of the main sufficient conditions for the intermediate solution of high and low levels of CPV against mother and father in adolescence.

Frequency Cut-Off: 1	High Levels of CPV Against the Mother Consistency Cut-Off: 0.82			Low Levels of CPV Against the Mother Consistency Cut-Off: 0.88	High Levels of CPV Against the Father Consistency Cut-Off: 0.81			Low Levels of CPV Against the Father Consistency Cut-Off: 0.85	
	1	2	3	1	1	2	3	1	2
Older				●				●	●
Women	●	●	●	●	●	●	●	●	●
Physical violence received			○		●		●		○
Verbal–emotional violence received	●	●		○	●			○	●
Psychopathy		●	●	○		●	●	○	○
Antisocial and law-violating behaviors	●		●	○		●		○	
Raw coverage	0.46	0.41	0.41	0.55	0.40	0.55	0.55	0.52	0.30
Unique coverage	0.09	0.04	0.13	0.55	0.09	0.08	0.05	0.31	0.09
Consistency	0.82	0.85	0.85	0.84	0.81	0.80	0.82	0.81	0.82
Overall solution consistency			0.79	0.84			0.76		0.79
Overall solution coverage			0.63	0.55			0.72		0.61

Note: ●: presence of condition, ○: absence of condition; For high levels of CPV against mother and father: 0, 0, 1, 1, 1, 1; for low levels of CPV against mother and father: 1, 1, 0, 0, 0, 0.

## Data Availability

The data are not publicly available because they are part of a larger research project, with more authors involved. However, data from the study can be made available upon reasoned request to the corresponding author.
